# Factor analytic mixed models for the provision of grower information from national crop variety testing programs

**DOI:** 10.1007/s00122-014-2412-x

**Published:** 2014-10-19

**Authors:** Alison B. Smith, Aanandini Ganesalingam, Haydn Kuchel, Brian R. Cullis

**Affiliations:** 1 National Institute for Applied Statistics Research Australia, University of Wollongong, Wollongong, Australia; 2 University of Western Australia, Perth, Australia; 3 Australian Grain Technologies, Roseworthy, Australia; 4 Computational Informatics, CSIRO, Canberra, Australia

## Abstract

****Key message**:**

**Factor analytic mixed models for national crop variety testing programs have the potential to improve industry productivity through appropriate modelling and reporting to growers of variety by environment interaction.**

**Abstract:**

Crop variety testing programs are conducted in many countries world-wide. Within each program, data are combined across locations and seasons, and analysed in order to provide information to assist growers in choosing the best varieties for their conditions. Despite major advances in the statistical analysis of multi-environment trial data, such methodology has not been adopted within national variety testing programs. The most commonly used approach involves a variance component model that includes variety and environment main effects, and variety by environment ($V\times E$) interaction effects. The variety predictions obtained from such an analysis, and subsequently reported to growers, are typically on a long-term regional basis. In Australia, the variance component model has been found to be inadequate in terms of modelling $V\times E$ interaction, and the reporting of information at a regional level often masks important local $V\times E$ interaction. In contrast, the factor analytic mixed model approach that is widely used in Australian plant breeding programs, has regularly been found to provide a parsimonious and informative model for $V\times E$ effects, and accurate predictions. In this paper we develop an approach for the analysis of crop variety evaluation data that is based on a factor analytic mixed model. The information obtained from such an analysis may well be superior, but will only enhance industry productivity if mechanisms exist for successful technology transfer. With this in mind, we offer a suggested reporting format that is user-friendly and contains far greater local information for individual growers than is currently the case.

## Introduction

In many countries it has long been the practice for plant breeding companies to submit potential new crop varieties for evaluation in series of field trials conducted by independent bodies. In Australia the system is known as the National Variety Trials (NVT), and is funded by the Australian Government and growers through the Grains Research and Development Corporation (GRDC) and managed by the Australian Crop Accreditation System (ACAS) Limited. NVT is a national program of comparative variety testing in which current commercial varieties and breeding lines that are very close to commercial release are evaluated. Over 600 trials are conducted annually and cover a range of crops including wheat, barley, canola, chick peas, faba beans, field peas, lentils, lupins, oats and triticale. In the United Kingdom (UK) there is a two-tiered testing system. Varieties are first tested for 2 years in the National List (NL) trials after which they are assessed for acceptance onto the NL. This is a legal requirement for marketing, and is aimed at ensuring that new varieties are genuinely new, that is they are distinct, uniform and stable (DUS) and have satisfactory value for cultivation and use (VCU). The NL process is administered by the Food and Environment Research Agency within the UK government Department for Environment, Food and Rural Affairs. Varieties accepted onto the NL may then be selected for evaluation in regional Recommended List (RL) trials for the provision of grower information. These trials are administered by the Home Grown Cereals Authority (HGCA) which is a division of the Agriculture and Horticulture Development Board and is funded by growers, dealers and processors in the cereals and oilseeds supply chain. NL systems are enforced in European Community countries, so that, for example, Germany and France have systems to assess DUS and VCU that are similar to the UK. Additionally they both have post-listing trials that are regionally based and aimed at providing grower information.

The importance of the information provided to growers by these national crop variety testing (CVT) programs cannot be underestimated in terms of grower and industry profitability, and potentially world food security. This is reflected in the level of investment by the relevant funding bodies. In Australia, the total annual investment by the GRDC for the NVT program is approximately AUD$5.5 m. In the UK, the RL trials are HGCA’s single biggest research project.

A key to maximising profitability and food security is the provision of information that is both accurate and of a sufficiently small scale to allow individual growers to choose the best varieties for their particular needs and environment. A critical factor in providing accurate information to growers is the use of appropriate methods for data analysis and reporting. The data relates to a large number of designed field experiments that cover a range of geographic locations and seasons. The complete set is known as a multi-environment trial (MET). Often, several traits are measured in these experiments, but the primary trait and the one under consideration in this paper is grain yield.

In Australia, the approach used for the analysis of NVT grain yield data has, until very recently, followed that described in Smith et al. (2001a). The MET analysis is accomplished using two stages. In the first stage, variety means are obtained from the separate analyses of individual trials. Also obtained from these analyses are statistical weights that provide a measure of uncertainty for the means. The weights are a function of numerous factors such as replication, design efficiency and error variance. The variety means from the first stage are then combined across trials to provide data for an overall mixed model analysis. Welham et al. (2010) show that although a one-stage analysis of individual plot data provides the most accurate variety predictions, the two-stage approach can work well for MET data when the variety $F$ tests for individual trials achieve a relatively high level of significance, and provided that weights are carried through to the second stage mixed model analysis. The latter is particularly important when there is substantial heterogeneity of within-trial error variances. Similar conclusions were reached in Mohring and Piepho (2009) and Piepho et al. (2012).

The second stage linear mixed model presented in Smith et al. (2001a) is a variance component model that includes (random) variety ($V$) main effects, (fixed) environment main effects and (random) variety by environment ($V\times E$) interaction effects. The cereal growing areas of Australia are divided into a number of geographic regions that have a historical basis and are used for the reporting of variety information. The $V\times E$ interaction effects in the mixed model are therefore partitioned into components including variety by region ($V\times R$), variety by year ($V\times Y$) and variety by region by year ($V\times R\times Y$) interaction. If trial locations are reasonably consistent from year to year, there may be a further partitioning into variety by location within region ($V\times L(R)$) interaction. The information reported annually to growers via the NVT Online web-site (ACAS 2007) includes the results of the individual trial analyses for that year and long-term variety predictions for each region as obtained from the MET analysis using the variety main effects and $V\times R$ [and $V\times L(R)$, where appropriate] interaction effects.

In the Australian context, it is well known that the variance component model is inadequate for modelling $V\times E$ interaction and that long-term regional means do not provide adequate information for growers. The latter may be seen if the interactions in the model are categorised as either “static” [$V\times R$ and $V\times L(R)$ interaction] or “non-static” (interactions involving varieties and years). Cullis et al. (2000) presented the analysis of 22 long-term yield data-sets related to a range of crop types from state-based testing programs. They showed that $V\times E$ interaction variance was large relative to $V$ main effect variance, accounting, on average, for 82 % of total genetic variance (the sum of the $V$ and $V\times E$ variances). The majority (95 %, on average), of $V\times E$ variance was attributed to non-static interaction. Grower information is based purely on the static effects with the non-static (seasonal) effects being ignored completely. The clear implication is that it is inadequate to use regional variety means for grower decisions since this disregards a large proportion of the total $V\times E$ interaction. This will be considered further in the “Discussion”.

It is difficult to determine exact details of the methodology used in other countries, but it is clear that all use a two-stage approach, with the second stage comprising a variance component model that includes variety and environment main effects, (either as fixed or random effects), and random $V\times E$ interaction effects, possibly partitioned in some way. Few countries seem to use weights in their second stage analysis. The reporting of results appears to follow a similar format to Australia. In the UK, the information reported by HGCA to growers via their web-site (AHDB 2013) includes the results of the individual trial analyses and long-term variety predictions from the MET analysis, both for the UK as a whole and on a regional basis.

The methodology used in most countries dates back to the comprehensive study of the UK variety testing system presented in Patterson and Silvey (1980). Much research into the analysis of MET data, in particular with an emphasis on superior models for $V\times E$ interaction, has been published since that time (see, for example Piepho 1997, 1998; Nabugoomu et al. 1999; Smith et al. 2001b, 2005; Theobald et al. 2002; Beeck et al. 2010; Cullis et al. 2010) and yet none has been implemented within the context of national crop variety evaluation programs. The reasons for this are unclear but may include statutory reasons or difficulties in implementing change given the number and diversity of stake-holders.

In Australia there has been a stark contrast in the methods used for the analysis of NVT and plant breeding MET data. Unlike the NVT scenario, the analysis of plant breeding data has progressed from the simple variance component model which is known to be inadequate in terms of modelling $V\times E$ interaction. Instead, most major Australian plant breeding programs use the factor analytic (FA) mixed model approach of Smith et al. (2001b). The FA mixed model for plant breeding data has been found to perform extremely well in terms of providing a parsimonious and informative model for $V\times E$ interaction and accurate predictions of variety effects.

A natural step forward for NVT, therefore, was to develop methodology based on the FA mixed model. This paper describes the results of this research. There were numerous hurdles to overcome in translating the FA mixed model of Smith et al. (2001b) for plant breeding MET data into the NVT setting. Statistical issues were largely related to differences in the structure of the data. In the plant breeding setting there are far less trials than in NVT and far more varieties. Furthermore, there are typically many more varieties than trials, but this is not the case in NVT. NVT data has historically, and for practical reasons, involved a two-stage approach for analysis. Plant breeding trials have fewer replicates than NVT so that, typically, a one-stage approach is necessary for the MET analysis (Welham et al. 2010). In addition to the statistical issues there are implementation issues, in particular associated with the reporting of results. In the season just concluded, the FA modelling approach presented in this paper was used for the analysis of NVT data for all crops tested. However the reporting of results remained at a regional level, thereby negating the benefits of using the FA approach. In this paper we propose alternative reporting formats.

## Statistical methods

As discussed in the “Introduction”, the analysis of NVT MET data is accomplished via a two-stage approach. The approach for individual trial analysis (the first stage), including the calculation of weights, is documented in Smith et al. (2001a). We assume that the (second stage) data relates to $t$ trials and a total of $m$ varieties and let $\mathbf {y}$ denote the $n \times 1$ combined vector of variety means from the analyses of individual trials. Typically the data are unbalanced, since not all varieties are grown in all trials, so that $n < < mt$. The second stage mixed model can be written as

1 $$\begin{aligned} \mathbf {y} = \mathbf {X}\varvec{\tau } + \mathbf {Z}\mathbf {u} + \mathbf {Z_p}\mathbf {u_p} + \varvec{\eta } \end{aligned}$$

where $\varvec{\tau }$ is a vector of fixed effects with associated design matrix $\mathbf {X}$; $\mathbf {u}$ is the $mt \times 1$ vector of random variety effects for each environment (ordered as varieties within environments) and has associated design matrix $\mathbf {Z}$; $\mathbf {u_p}$ is a vector of random non-genetic (peripheral) effects with associated design matrix $\mathbf {Z_p}$ and $\varvec{\eta }$ is a vector of effects that accounts for the fact that the data comprise estimates and are therefore subject to uncertainty. Note that typically $\varvec{\tau }$ is simply the $t \times 1$ vector of trial means and $\mathbf {u_p}$ is omitted.

We assume that $\mathbf {u}$, $\mathbf {u_p}$ and $\varvec{\eta }$ are mutually independent, and distributed as multivariate Gaussian, with zero means. The variance matrix for $\mathbf {u_p}$ is given by $\mathbf {G_p} = \oplus _{k=1}^b \sigma ^2_{p_k} \mathbf {I}_{q_k}$ where $b$ is the number of components in $\mathbf {u_p}$ and $q_k$ is the number of effects in (length of) $\mathbf {u_p}_k$. The variance matrix for $\varvec{\eta }$ is assumed known from the first stage and is given by $\varvec{\Sigma } = \oplus _{j=1}^t \varvec{\Pi }^{-1}_j$ where $\varvec{\Pi }^{-1}_j$ is a diagonal matrix with elements given by the weights for trial $j$. We assume that the variance matrix of the variety effects is given by

2 $$\begin{aligned} \mathrm{var}\left( \mathbf {u}\right) = \mathbf {G}_e \otimes \mathbf {I}_m \end{aligned}$$

where $\mathbf {G_e}$ is a $t \times t$ symmetric positive (semi)-definite matrix that is often referred to as the between environment genetic variance matrix.

It is of interest to consider several forms for $\mathbf {G_e}$. The first is a diagonal form, namely $\mathbf {G_e} = \oplus _{j=1}^t \sigma ^2_{g_j}$ where $\sigma ^2_{g_j}$ is the genetic variance for environment $j$. In this variance structure the variety effects are assumed independent between environments so there is an analogy with the separate analyses of individual trials. The simplest model that accommodates correlations between variety effects in different environments is the compound symmetric form which arises by assuming a model for $\mathbf {u}$, namely

3 $$\begin{aligned} \mathbf {u} = \mathbf {Z_g} \mathbf {u_g} + \mathbf {u_{ge}} \end{aligned}$$

where $\mathbf {u_g}$ is the $m \times 1$ vector of variety main effects with variance matrix $\sigma ^2_g \mathbf {I}_m$ and $\mathbf {u_{ge}}$ is the $mt \times 1$ vector of variety by environment interaction effects with variance matrix $\sigma ^2_{ge} \mathbf {I}_{mt}$. The design matrix for the main effects is given by $\mathbf {Z_g} = \left( \mathbf {1}_t \otimes \mathbf {I}_m\right)$ so that $\mathbf {G_e} = \sigma ^2_g\mathbf {J}_t + \sigma ^2_{ge}\mathbf {I}_t$, where $\mathbf {J}_t$ is a $t \times t$ matrix in which all elements are unity.

The model in Eq. (3) is a variance component model, since all random terms have variance matrices that are scaled identity matrices. It is a very restrictive model since it leads to the assumption that the genetic variance is the same for all environments, and is given by $\sigma ^2_g + \sigma ^2_{ge}$, and the genetic covariance for all pairs of environments is $\sigma ^2_g$. Often, more general variance component models are used in which the variety by environment interaction effects are partitioned further, for example into variety by year, variety by region, variety by year by region and residual variety by environment effects. Such a model was used by Smith et al. (2001a). Even with this partitioning the resultant form for $\mathbf {G_e}$ is over-simplified and rarely provides a good fit to the data.

The most general model for $\mathbf {G_e}$ is the unstructured form that contains $p = t(t+1)/2$ parameters to be estimated, namely a genetic variance for each environment and covariance between each pair of environments. Clearly as the number of trials increases, the number of parameters becomes prohibitively large and this influences both the ability to fit the model and to reliably estimate the variance parameters. The unstructured model is therefore rarely used for the analysis of MET data.

In the context of one-stage analyses of plant breeding MET data, we have found that the FA variance model (Smith et al. 2001b) provides a good approximation to the unstructured form (Kelly et al. 2007) and is both parsimonious and illuminating. The aim of the FA model as applied to the variety effects in different environments is to account for the genetic covariances between environments in terms of a small number of hypothetical factors. The number of factors is called the order of the model and we let FAk denote an FA model of order $k$. The FAk model for the effect of variety $i$ in environment $j$ can be written as

4 $$\begin{aligned} u_{ij} = \lambda _{1j}f_{1i} + \lambda _{2j}f_{2i} + \cdots + \lambda _{kj}f_{ki} + \delta _{ij} \end{aligned}$$

where $f_{ri}$ is the value (also called a score) of the $r$th hypothetical factor ($r = 1, \ldots , k$) for variety $i$ and $\lambda _{rj}$ is the coefficient (also called a loading) for environment $j$. The factors are usually assumed to be independent with unit variance so that $\mathrm{var}\left( f_{ri}\right) = 1$. The model can also be viewed as a multiple regression of the variety effects for an environment on a set of environmental covariates (loadings) with a separate slope (score) for each variety (also see Burgueno et al. 2008). The feature which distinguishes the FA model from an ordinary regression is that not only are the slopes estimated from the data, but also the covariates. The final term $\delta _{ij}$ represents the lack of fit of the regression so will be termed a genetic regression residual. The model in Eq. (4) can be written in vector notation as

5 $$\begin{aligned} \mathbf {u} = \left( \varvec{\Lambda } \otimes \mathbf {I}_m \right) \mathbf {f} + \varvec{\delta } \end{aligned}$$

where $\varvec{\Lambda }$ is the $t \times k$ matrix of loadings, $\mathbf {f}$ is the $mk \times 1$ vector of scores and $\varvec{\delta }$ is the $mt \times 1$ vector of genetic regression residuals. The vectors of random effects $\mathbf {f}$ and $\varvec{\delta }$ are assumed to be mutually independent and distributed as multivariate Gaussian with zero means. The variance matrices are assumed to be $\mathrm{var}\left( \mathbf {f}\right) = \mathbf {I}_{mk}$ and $\mathrm{var}\left( \varvec{\delta }\right) = \varvec{\psi } \otimes \mathbf {I}_m$ where $\varvec{\psi }$ is a $t \times t$ diagonal matrix with a variance (called a specific variance) for each environment. Finally, these assumptions lead to a variance for $\mathbf {u}$ given by

6 $$\begin{aligned} \mathrm{var}\left( \mathbf {u}\right) = \left( \varvec{\Lambda }\varvec{\Lambda }^{\!\scriptscriptstyle \top }+ \varvec{\psi }\right) \otimes \mathbf {I}_m \end{aligned}$$

so that the between environment genetic variance matrix is $\mathbf {G_e} = \left( \varvec{\Lambda }\varvec{\Lambda }^{\!\scriptscriptstyle \top }+ \varvec{\psi }\right)$.

In this paper we propose the use of FA models for variety by environment effects in two-stage analyses of crop variety evaluation data.

### FA model fitting and tools for interpretation

All models in this paper were fitted using the ASReml-R package (Butler et al. 2009) within R (R Core Team 2013). The variance parameters in the mixed model of Eq. (1) are estimated using residual maximum likelihood (REML). In terms of the FA model, the variance parameters are the loadings and specific variances and the REML estimates of these will be denoted by $\hat{\lambda }_{rj}$ and $\hat{\psi }_j$ ($r = 1, \ldots , k; j = 1, \ldots , t$). Note that when $k>1$, the loading matrix $\varvec{\Lambda }$ is not unique so that estimation necessitates the imposition of constraints. The algorithm in ASReml-R (Butler et al. 2009) fixes all $k(k-1)/2$ elements in the upper triangle of $\varvec{\Lambda }$ to zero. Once an estimate of $\varvec{\Lambda }$ has been obtained, the matrix may be rotated as desired for interpretative purposes (see below).

Given estimates of all the variance parameters, we obtain empirical best linear unbiased estimates of the fixed effects and empirical best linear unbiased predictions (EBLUPs) of the random effects. In terms of the FA model we denote the EBLUPs of the factor scores and genetic regression residuals by $\tilde{f}_{ri}$ and $\tilde{\delta }_{ij}$ ($r = 1, \ldots , k; i = 1, \ldots , m; j = 1, \ldots , t$).

The model fitting process commences with the fitting of an FA1 model, then proceeds to higher order models as necessary. An appropriate order may be determined using likelihood based measures that compare sequences of FA models. Since such models are nested, residual maximum likelihood ratio tests (REMLRT) can be used, but so too can information criteria such as the Akaike and Bayesian information criteria (AIC and BIC, respectively). In our experience the application of REMLRT and AIC tend to lead to the selection of very high order models that are unnecessarily complicated. In contrast, the application of BIC which emphasises parsimony, leads to the choice of models that may underfit. A superior approach for the selection of an appropriate order may involve the comparison between an FAk model and the unstructured model, but since the latter typically cannot be fitted, an alternative type of test statistic would be required. This is the subject of current research. In the absence of such a test we choose to use a pragmatic approach based on a goodness-of-fit measure similar to that used for a standard multiple regression. We therefore compute the percentage of genetic variance accounted for by the $k$ factors, both for individual environments (denoted $v_j$) and overall (denoted $\bar{v}$):

$$\begin{aligned} v_{j}&=\left. 100 \sum _{r=1}^k \hat{\lambda }^2_{rj} \right/\left( \sum _{r=1}^k \hat{\lambda }^2_{rj} + \hat{\psi }_j \right) \\ \bar{v}&=\left. 100\mathrm{tr}\left( \hat{\varvec{\Lambda }}\hat{\varvec{\Lambda }}^{\!\scriptscriptstyle \top }\right) \right/\mathrm{tr}\left( \hat{\varvec{\Lambda }}\hat{\varvec{\Lambda }}^{\!\scriptscriptstyle \top }+ \hat{\varvec{\psi }}\right) \end{aligned}$$

where the operator “$\mathrm{tr}\left( \right)$” computes the trace of the matrix argument. The order of FA model may then be chosen on the basis of both the overall percentage accounted for and the distribution of individual environment values, since it is desirable for the chosen model to have few environments with low values and many environments with high values.

The fitting of an FA model provides the REML estimate of the between environment genetic variance matrix as $\hat{\mathbf {G}}_{\mathbf {e}} = \left( \hat{\varvec{\Lambda }}\hat{\varvec{\Lambda }}^{\!\scriptscriptstyle \top }+ \hat{\varvec{\psi }}\right)$. This can be converted to a correlation matrix, $\hat{\mathbf {C}}_{\mathbf {e}} = \hat{\mathbf {D}}_{\mathbf {e}}\hat{\mathbf {G}}_{\mathbf {e}}\hat{\mathbf {D}}_{\mathbf {e}}$, where $\hat{\mathbf {D}}_{\mathbf {e}}$ is a diagonal matrix with elements given by the inverse of the square roots of the diagonal elements of $\hat{\mathbf {G}}_{\mathbf {e}}$. Investigation of this matrix will reveal variety by environment interaction in the sense of pairs of environments that have low, or possibly even negative estimated genetic correlations. In such cases the rankings of the varieties will differ substantially between the environments and this is likely to be important information for growers. The matrix $\hat{\mathbf {C}}_{\mathbf {e}}$ has dimension $t \times t$, so, for large values of $t$ we choose to display $\hat{\mathbf {C}}_{\mathbf {e}}$ graphically, using a heatmap in R (R Core Team 2013), re-ordering the rows and columns to aid with visualisation. In this paper we have chosen to order on the basis of the dendrogram obtained using the agnes package (an agglomerative hierarchical clustering algorithm) in R (R Core Team 2013) with $\mathbf {I}_t - \hat{\mathbf {C}}_{\mathbf {e}}$ as the dissimilarity matrix. In this way, environments that are highly correlated (so exhibit little cross-over interaction) are located close together on the heatmap, whereas less well correlated environments will be further apart.

In terms of variety predictions from the FA model, we can compute the EBLUP of the effect of variety $i$ in environment $j$ as

7 $$\begin{aligned} \tilde{u}_{ij}&= \hat{\lambda }_{1j}\tilde{f}_{1i} + \hat{\lambda }_{2j}\tilde{f}_{2i} + \cdots + \hat{\lambda }_{kj}\tilde{f}_{ki} + \tilde{\delta }_{ij} \nonumber \\&= \tilde{\beta }_{ij} + \tilde{\delta }_{ij} \end{aligned}$$

where $\tilde{\beta }_{ij}$ is the predicted regression component. The regression component is based purely on the underlying factors so represents the variety by environment variation that has repeatability in terms of the data under study and with reference to the FA model fitted. In contrast, the genetic regression residuals represent non-repeatable variety effects, that is, effects which are specific to individual environments, given the model and set of environments. In terms of variety information for growers we therefore choose to use the predicted regression component $\tilde{\beta }_{ij}$ rather than the full predicted effect $\tilde{u}_{ij}$ (see Cullis et al. 2010 for a full discussion). This has two important consequences. The first is that we obtain compatible predictions of variety effects for every environment, irrespective of whether the variety was grown in the environment. The second is that we must be wary of variety predictions for those environments where the percentage of variance accounted for by the regression is low.

The regression form of the variety predictions from an FA model allows investigation of variety stability in terms of responses to changes in environment, for those environments observed in the data. Each factor score, for $r = 1, \ldots , k$, in Eq. (7) reflects the response of that individual to the corresponding environmental covariate (loading). If these are to be interpreted individually as stabilities, and if $k>1$, it is usually most meaningful to rotate the estimated loadings (which have been constrained for estimation) to a principal component solution (Cullis et al. 2010). In this case the first rotated factor accounts for the maximum amount of genetic covariance in the data, the second accounts for the next largest amount and is orthogonal to the first, and so on. We denote the rotated estimated loadings and scores by $\hat{\lambda }^*_{ij}$ and $\tilde{f}^*_{ij}$ so that $\tilde{\beta }_{ij}$ from Eq. (7) can now be written as $\sum _{r=1}^k \hat{\lambda }^*_{rj}\tilde{f}^*_{ri}$. The multiple regression in terms of the rotated factors can then be displayed graphically, for an individual variety, using so-called latent regression plots which are similar to added variable plots with the advantage that there is a natural ordering of the variables. We may therefore construct $k$ plots for variety $i$, with the $y$- and $x$-axes for the first plot corresponding to $\tilde{\beta }_{ij}$ and $\hat{\lambda }^*_{1j}$ respectively. The points on this plot are located about a line that has slope given by $\tilde{f}_{1i}$ so we add this line to the plot. Subsequent plots adjust the $y$- and $x$-axes for preceding factors. Thus the $y$-axis for plot $s$ ($s =2, \ldots , k$) corresponds to $\tilde{\beta }_{ij} - \sum _{r=1}^{s-1} \hat{\lambda }^*_{rj}\tilde{f}^*_{ri}$ and the $x$-axis to $\hat{\lambda }^*_{sj}$. The line drawn on plot $s$ ($s =2, \ldots , k$) for variety $i$ has slope given by $\tilde{f}^*_{si}$.

Finally we propose that the variety predictions be accompanied by a measure of accuracy. Any such measure will be based on the prediction error variance (PEV) matrix, which, for the complete vector of predictions, $\tilde{\varvec{\beta }}$, is given by

8 $$\begin{aligned} \mathbf {V}_{\beta } = \mathrm{var}\left( \tilde{\varvec{\beta }} - \varvec{\beta }\right) = \left( \hat{\varvec{\Lambda }}^{*} \otimes \mathbf {I}_m \right) \mathbf {V}_{f^{*}} \left( \hat{\varvec{\Lambda }}^{*{\!\scriptscriptstyle \top }} \otimes \mathbf {I}_m \right) \end{aligned}$$

where $\mathbf {V}_{f^*} = \mathrm{var}\left( \tilde{\mathbf {f}}^{*} - \mathbf {f}^{*}\right)$ is the PEV matrix for the rotated scores. Note that we could equally have used the PEV matrix for the unrotated scores, which could be obtained directly from the fit of the mixed model, but the accuracy of the rotated scores themselves is of interest given their interpretation as indicators of varietal stability. The computation of the PEV matrix for the rotated scores requires an additional iteration of model fitting in ASReml-R in which the rotated REML estimates of the loadings are incorporated. Thence the EBLUPs of the variety scores from this fit of the model are on the rotated scale. Details are available from the authors on request. Finally, we note that the formulation of the PEV matrix in Eq. (8) ignores any uncertainty in the estimation of the variance parameters, $\hat{\varvec{\Lambda }}^*$.

## Motivating example

We apply the new method of analysis to yield data from NVT wheat trials for the Southern mega-region (see next section) for the five year period 2009–2013. The data-set comprised 196 trials and 200 varieties. Since formation of an appropriate data-set is crucial for both the accuracy and relevance of the resultant variety predictions we commence by discussing this in detail.

### Description of data

NVT has been in operation since 2005 so there is potential to conduct analyses using data that spans a 9 year period (2005–2013) and all growing regions across Australia. In the case of wheat, the crop to be investigated here, this amounts to a total of 1,086 trials. However there are various reasons why a reduced data-set is used for analysis. The key drivers of deciding which trials to include in the data-set are the need to obtain a representative sample of environments, both in a geographic and seasonal sense, a relevant set of varieties and reasonable connectivity (number of varieties in common) between pairs of trials. Thus there is a trade-off in achieving the first aim compared with the latter two since the first requires a data-set that is as broad and long as possible, whereas the latter two require a judicious narrowing of the scope of the data.

**Fig. 1:**
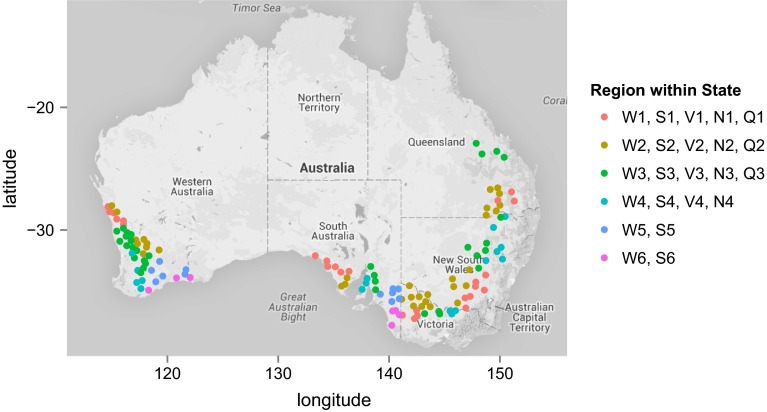
Map of Australia showing location of 2013 wheat trials, with regions within states differentiated by *colour*

In order to illustrate the geographic issues, we consider the most recent year of data, 2013, in which 129 (main season) wheat trials were harvested. The wheat growing areas of Australia are divided into 23 regions that have a historical and intuitive basis and are still used for the reporting of variety information to growers. This aspect will be discussed in some detail in later sections of the paper. There are six regions in the state of Western Australia (which will be labelled as W1–W6), six in South Australia (S1–S6), four in Victoria (V1–V4), four in New South Wales (N1–N4) and three in Queensland (Q1–Q3). The locations of the 2013 trials, together with their regions, are shown in Fig. 1.

**Fig. 2:**
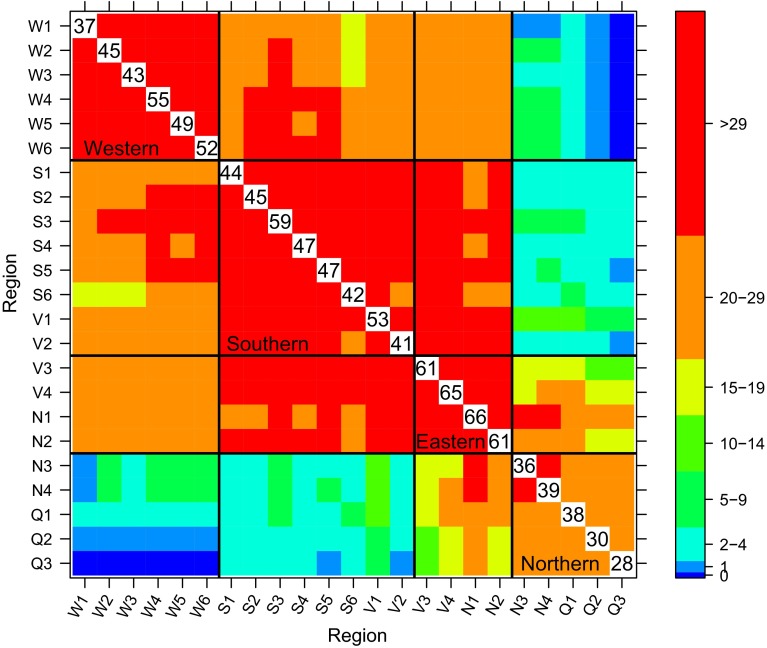
Variety connectivity across regions for the 2013 wheat trials. The *numbers along the diagonal* are the average number of varieties grown in a trial for each region and the *colours of the boxes on the off-diagonals* indicate the average number of varieties in common between pairs of trials in different regions. Boundaries for mega-regions are also indicated

Figure 1 reveals that wheat is grown over a wide area in Australia. There is a large range in climate and soil characteristics across this landscape and there are diverse management practices. As a consequence, varieties are usually bred for specific adaptation and this is reflected in the NVT program, with the majority of varieties only being tested in their target environments. Figure 2 shows the connectivity between trials in 2013 on a regional basis. The stand-out feature of this figure is that very few of the varieties grown in Western Australia are grown in Queensland (the average number in common between regions W1–W6 and Q1–Q3 is 0, 1 or 2) or northern New South Wales (the average number in common between W1–W6 and N3, N4 is always less than 9) and vice versa.

The poor connectivity shown for many of the cells in Fig. 2 may have an impact on the statistical analysis, in particular the reliability of estimation of $\mathbf {G_e}$. With an unstructured form for $\mathbf {G_e}$, the parameters to be estimated are a genetic variance for each environment and a covariance for each pair of environments. The amount of information for estimating a covariance is dependent on the number of varieties in common between the pair of trials concerned, so it would not be possible to fit an unstructured model for $\mathbf {G_e}$ for data exhibiting the pattern of connectivity as in Fig. 2. The situation is more complex for FA models since the underlying factors provide links between trials, but pairwise connectivity is still likely to be important for the estimation of the variance parameters in $\varvec{\Lambda }$. This is the subject of current research. Certainly experience has shown that if a subset of trials is completely disconnected from the rest then FA models can not be successfully fitted. In Fig. 2 there are no such subsets, rather there is a moving window of connectivity so that, for example, trials in Western Australia are indirectly connected with trials in Queensland via trials in intermediate regions. It may well be possible to fit FA models to such data but there may be doubt about the reliability of the resultant variance parameter estimates. Therefore, using the connectivity patterns across regions for each year as a guide, and in conjunction with expert agronomic advice from ACAS, the country was divided into four “mega-regions” for the purposes of analysis. The mega-regions were defined as Western (regions W1–W6), Southern (regions S1–S6 and V1 and V2), Eastern (regions V3, V4, N1 and N2) and Northern (regions N3, N4 and Q1–Q3). The boundaries for these mega-regions are also marked on Fig. 2. In the remainder of this paper we will focus attention on the Southern mega-region.

**Fig. 3:**
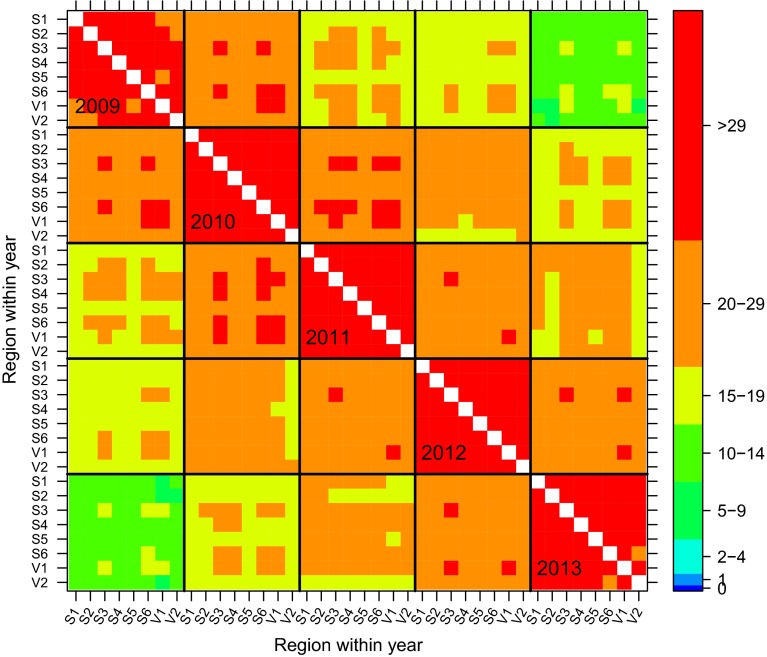
Variety connectivity across regions and the years 2009–2013 for the Southern mega-region. The *colours of the boxes on the off-diagonals* indicate the average number of varieties in common between pairs of trials in different regions and years. Boundaries for years are indicated

We now consider the variety connectivity issue in terms of time-span. The nature of the testing system is such that new varieties are added each year and this necessitates the removal of older varieties or those no longer of commercial interest. This means that connectivity between trials decreases as the separation in years increases. Although the retention rate varies slightly between regions, a 5 year time-span appears to ensure good connectivity for all regions, so has been used for the most recent NVT analysis. Figure 3 shows the connectivity between trials in the Southern mega-region for the period 2009–2013 on a region within year basis. The poorest connectivity, between trials in 2009 and 2013, is still quite reasonable, with the smallest average number of varieties in common being 8 (between S2 and V1) but greater than 10 for most pairs of regions.

All trials are included in the MET data-set unless they exhibit no genetic variance. Such trials can either be identified from the first stage analyses of individual trials, in which case they will have an $F$-ratio of less than 1 for the (fixed) variety effects, or from the MET analysis using a diagonal form for $\mathbf {G_e}$, in which case they will have an estimated genetic variance fixed at zero. All varieties in these trials are included in the data-set unless they were grown in less than 4 trials or were so-called “filler” varieties of no relevance to growers.

**Fig. 4:**
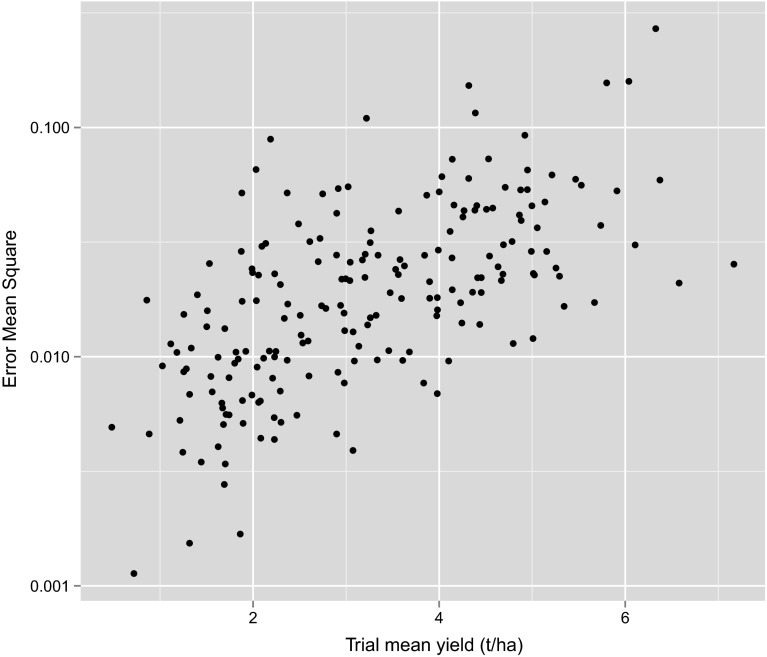
Trial mean yields and error mean squares from separate analyses of individual trials for 196 wheat trials in Southern mega-region between 2009 and 2013. Note the use of a log scale for the $y$-axis

The final data-set for the analysis of the Southern mega-region comprised 196 trials and 200 varieties. Figure 4 shows that there was a large range in both the trial mean yields and the error mean squares (obtained from first stage analyses as documented in Smith et al. 2001a). The over 200-fold difference between smallest and largest error mean squares highlights the need for using weights in the second stage MET analysis.

### Results of analysis

**Fig. 5:**
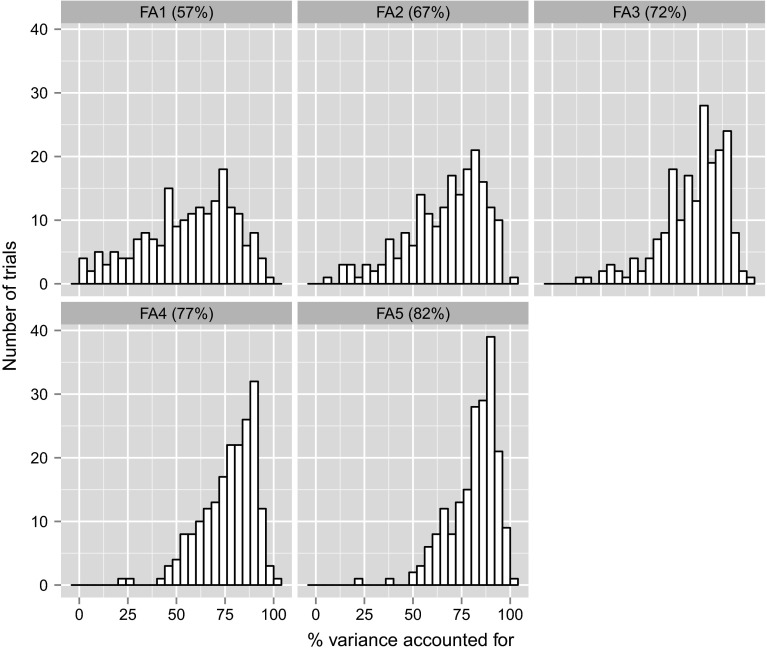
Distribution of percentage variance accounted for in FA models fitted to between environment genetic variance matrix. Overall percentage for each FA model is given in *parentheses*

Here we describe the new method of analysis for the Southern mega-region wheat MET dataset for the period 2009–2013. FA models were fitted to $\mathbf {G_e}$ until the overall percentage variance accounted for exceeded 80 %. This resulted in the fitting of models of orders 1 through 5. The distribution of the individual trial percentage variances accounted for, $v_j$, together with the overall value, $\bar{v}$, for each order, are shown in Fig. 5. The overall percentage variance accounted for by the FA5 model was 82 and 159 out of the 196 trials had an individual value greater than 70 %. We therefore chose the FA5 model as providing an adequate fit to the data. The application of AIC and REMLRT all showed the FA5 model to be superior to the lower order models (see Table 1). (Note that the $p$-values for the REMLRT comparing each pair of FA models were all less than 0.001). They also tended to suggest the need for fitting FA6 and possibly higher order models. Such models were attempted but there were computational difficulties that may have been due either to the connectivity in the data or to issues with the model-fitting algorithm. In contrast, the application of BIC would lead to the choice of the FA2 model. These inconsistencies support the use of the more pragmatic approach based on variance accounted for.

We also fitted a variance component model of the form historically used for Australian crop variety evaluation data. In this model the variety effects for each environment, $\mathbf {u}$, were modelled as the sum of variety main effects, variety by region, variety by year, variety by region by year and residual variety by environment interaction effects. Table 1 shows the inferiority of this model in terms of goodness-of-fit compared with any of the FA models. Additionally, we note that variety predictions from the variance component model are usually reported on a regional basis so are obtained as the sum of the variety main effects and variety by region interactions. Thus the sum of the variety main effect and variety by region interaction variance components as a percentage of the sum of all the variance components is analogous to the overall percentage of variance accounted for by an FA model. For the data under study this value was only 42, which is substantially lower than $\bar{v}$ for even the FA1 model. Note that this figure comprises 38 % from the variety main effect variance, and only 4 % for the variety by region variance. Thus the long-term regional predictions capture only a very small amount of $V\times E$ interaction. These findings are typical in the analysis of Australian crop variety evaluation data (Cullis et al. 2000).

**Table 1:** Summary of models fitted (diagonal, FA1–FA5 and variance component) to between environment genetic variance matrix: number of parameters in model, residual log likelihood, AIC and BIC and percentage of variance accounted for

Model	Parameters	Residual logl	AIC	BIC	% vaf
DIAG	196	7,051	$-$13,709	$-$12,311	
FA1	392	9,274	$-$17,764	$-$14,967	57
FA2	586	10,216	$-$19,260	$-$15,079	67
FA3	780	10,741	$-$19,922	$-$14,357	72
FA4	973	11,161	$-$20,375	$-$13,434	77
FA5	1,165	11,512	$-$20,694	$-$12,383	82
VC	5	7,074	$-$14,138	$-$14,103	42

**Fig. 6:**
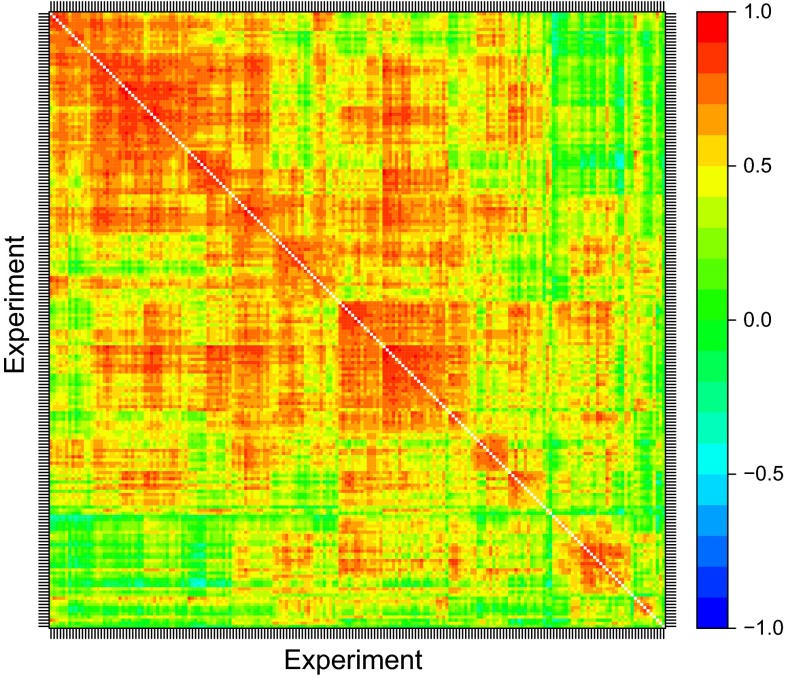
Heatmap of the estimated between environment genetic correlation matrix, ordered on the basis of a dendrogram

**Fig. 7:**
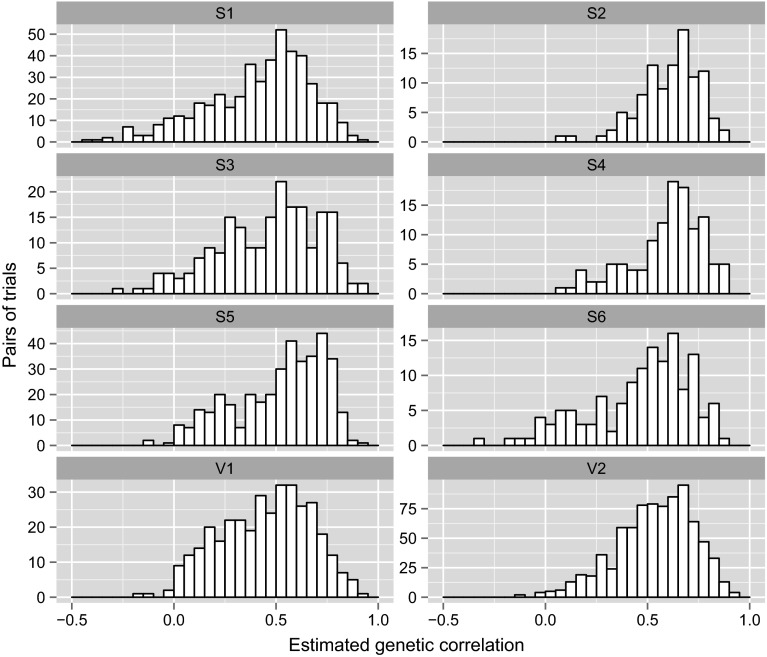
Distribution of estimated pairwise genetic correlations for trials in each region

The estimated between environment genetic correlation matrix from the FA5 model is displayed graphically in Fig. 6. The rows and columns have been ordered on the basis of a dendrogram as described in the statistical methods section. Figure 6 suggests there is structure in the correlations, with several large groups of trials within which the pairwise estimated correlations are all high. Thus all the trials within a group had similar rankings of varieties, whereas there were often substantial cross-overs of rankings for trials in different groups. The formation of such groups based on the dendrogram is purely exploratory and any interpretation requires the use of (external) environmental covariate information. The information currently available for NVT is inadequate for this purpose but research is aimed at compiling a more comprehensive set of covariates. Importantly, however, the groups observed on Fig. 6 do not co-incide with the geographic regions traditionally used for reporting. In the context of FA models, regional variety predictions could be obtained by averaging the predicted regression components, $\tilde{\beta }_{ij}$, across all trials in the region concerned. If this approach is adopted the averaging will include pairs of trials that are poorly (sometimes negatively) correlated (see Fig. 7) so that substantial cross-over variety by environment interaction would be ignored. Much of the cross-over interaction is between trials in different years although sometimes different locations within the regions.

**Fig. 8:**
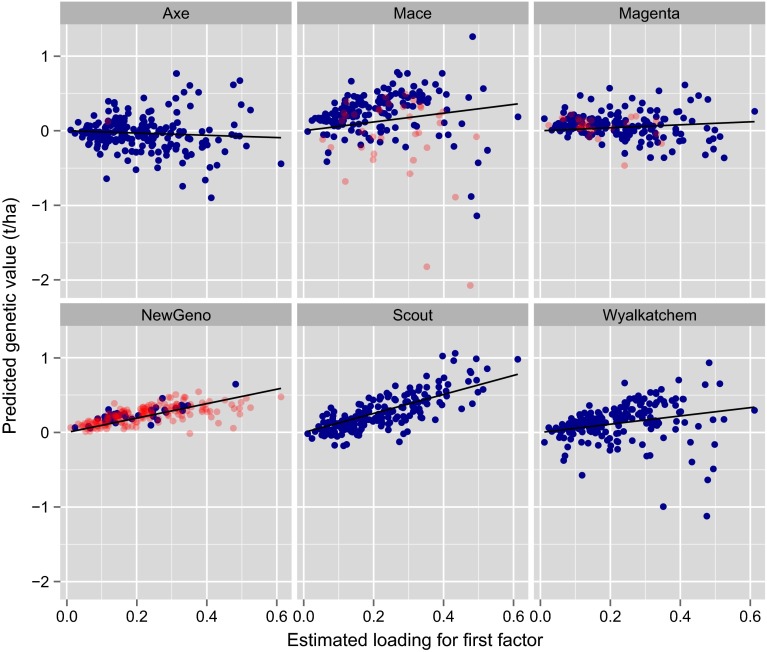
Latent regression plot for first factor for six genotypes. *Points are coloured blue/red* if genotype was grown/not grown in the associated trial. The *solid line* has slope given by the predicted score for the genotype for the first factor

**Fig. 9:**
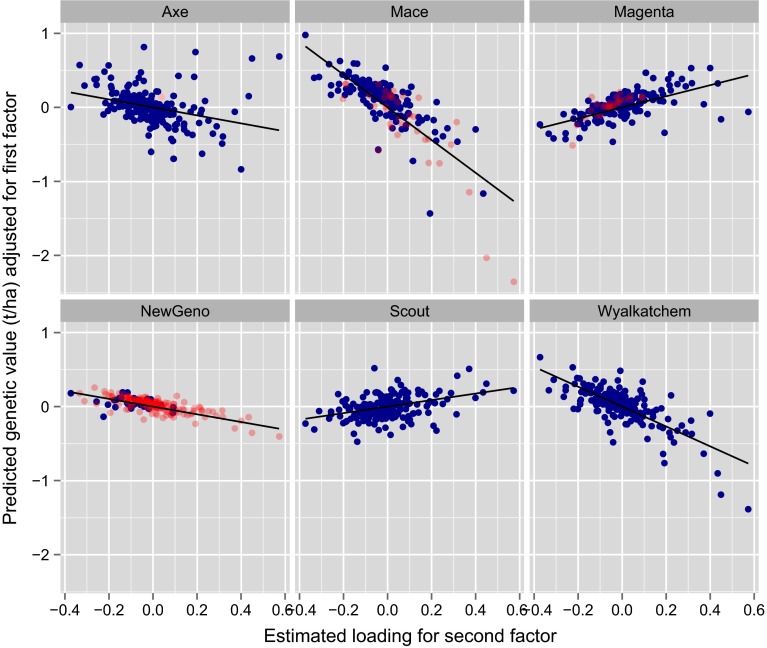
Latent regression plot for second factor for six genotypes. *Points are coloured blue/red* if genotype was grown/not grown in the associated trial. The *solid line* has slope given by the predicted score for the genotype for the second factor

**Fig. 10:**
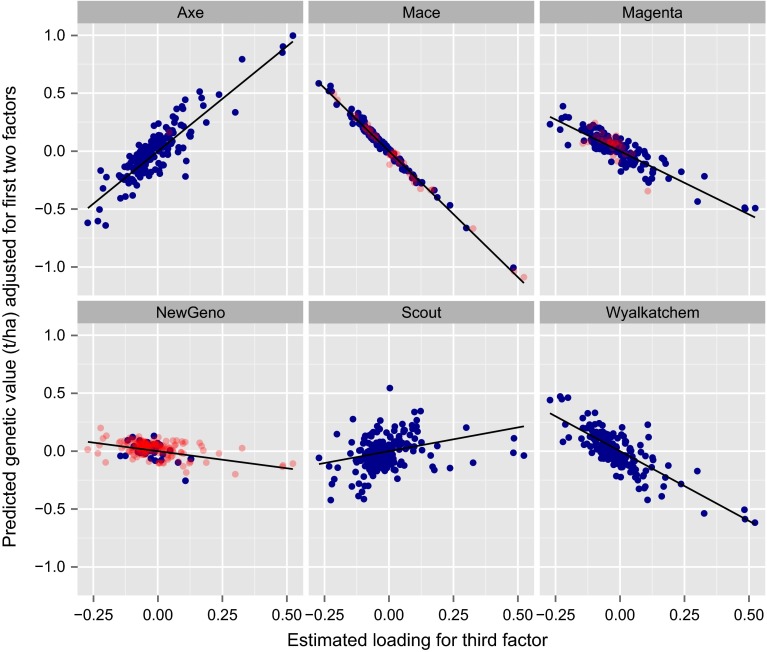
Latent regression plot for third factor for six genotypes. *Points are coloured blue/red* if genotype was grown/not grown in the associated trial. The *solid line* has slope given by the predicted score for the genotype for the third factor

Exploration of the estimated genetic correlation matrix may allow characterisation of environments according to their patterns of variety by environment interaction. In terms of grower information it is arguably more important to consider interaction from the complementary perspective of the varieties. Growers need to know how varieties of interest to them respond to changes in environment. The latent regression plots described previously provide one means for exploring this so-called variety stability. Recall that in order to aid with the interpretation, the loadings are rotated to a principal component solution. In the data under study, the rotated loadings accounted for 47, 12, 7, 7 and 6 % of the total genetic variation. Our interest focusses on five current commercial varieties (Axe, Mace, Magenta, Scout and Wyalkatchem) that are widely grown in the region, and a potential new variety (hence-forth called NewGeno) under consideration for commercial release. The latent regression plots for these six varieties and for the first three factors are given in Figs. 8, 9 and 10. The remaining plots have been excluded for reasons of brevity. The points on each plot are coloured blue if the variety was grown in the associated trial and red otherwise. The varieties Scout and Wyalkatchem were grown in all 196 trials in the data-set, Axe was grown in 195 trials and Mace and Magenta were grown in less trials (169 and 160 respectively), but in every year, and NewGeno was only grown in 2013 (a total of 38 trials). Recall that the lines on the latent regression plots have slopes given by the predicted genotype scores. These are given explicitly, together with their standard errors, in Table 2. In this study the first (rotated) factor accounted for the vast majority of variety by environment variation so that the regressions on this factor have the greatest impact on the predicted genetic values. Since all the estimated environment loadings for this factor are positive (see $x$-axis in Fig. 8), this then means that large positive slopes for this factor are desirable. Of the varieties listed in Table 2, Scout has the highest predicted score. Figure 8 shows that the genetic values for Scout are nearly always positive, and, as suggested by the regression, they increase substantially for environments with high estimated loadings. The points for Scout show less spread about the line than do most of the other varieties, suggesting the relative importance of the first factor for this variety. The picture conveyed in Fig. 8 is compatible with the commercial performance of Scout which is known to be a consistently high yielding variety across the Southern mega-region.

**Table 2:** Predicted (rotated) factor scores (with standard errors underneath) from the FA5 model for six genotypes

Genotype	Factor 1	Factor 2	Factor 3	Factor 4	Factor 5
Axe	$-$0.15	$-$0.54	1.81	$-$0.63	1.18
	0.0082	0.0156	0.0243	0.0216	0.0255
Mace	0.59	$-$2.21	$-$2.18	0.22	0.23
	0.0095	0.0205	0.0360	0.0234	0.0307
Magenta	0.20	0.75	$-$1.09	$-$0.04	0.81
	0.0085	0.0167	0.0257	0.0238	0.0303
NewGeno	0.97	$-$0.52	$-$0.30	$-$0.22	0.70
	0.0421	0.0997	0.2068	0.1225	0.0898
Scout	1.27	0.44	0.41	$-$1.00	$-$1.31
	0.0082	0.0157	0.0244	0.0216	0.0255
Wyalkatchem	0.55	$-$1.34	$-$1.21	0.88	$-$0.86
	0.0082	0.0156	0.0242	0.0216	0.0255

The variety Mace has a large negative response to the second and third factors (Figs. 9, 10). Similar, but slightly more moderate responses are observed for Wyalkatchem. These results are consistent with the fact that Mace was derived from a cross involving Wyalkatchem as a parent. The variety Axe, which is a much earlier maturing variety than the other varieties in Table 2, has a large positive response to the third factor (Fig. 10). The interpretation of factors can be difficult, and requires the use of environmental covariate information. As previously discussed, the information currently available for NVT is not sufficiently comprehensive. We note, however, that there was evidence of a relationship between the loadings for the first factor and trial mean yield (correlation of 0.73). In terms of the second and third factors, many of the trials with high loadings had been subjected to either frost or disease events, and many of the trials with low loadings were sown relatively early in the season. These aspects may help to explain the responses of Mace, Wyalkatchem and Axe.

The latent regression plots for the second and third factors for NewGeno are interesting in the sense that trials in which this variety were grown were limited to those with relatively low loadings. The resultant standard errors for the scores for these factors, in particular the third factor, are extremely large (Table 2). This alerts us to exercising caution when comparing the responses of NewGeno to the second and third factors with those of other varieties. In fact, we note that, according to pedigree information, NewGeno should exhibit similar responses to Mace. The observation about poor coverage of loadings (and thence large standard errors for factor scores) for some of the newer varieties leads to the conclusion that data from a wider range of environments is required in order to make confident statements about the stability of these varieties. Methods for achieving this are the subject of current research.

**Fig. 11:**
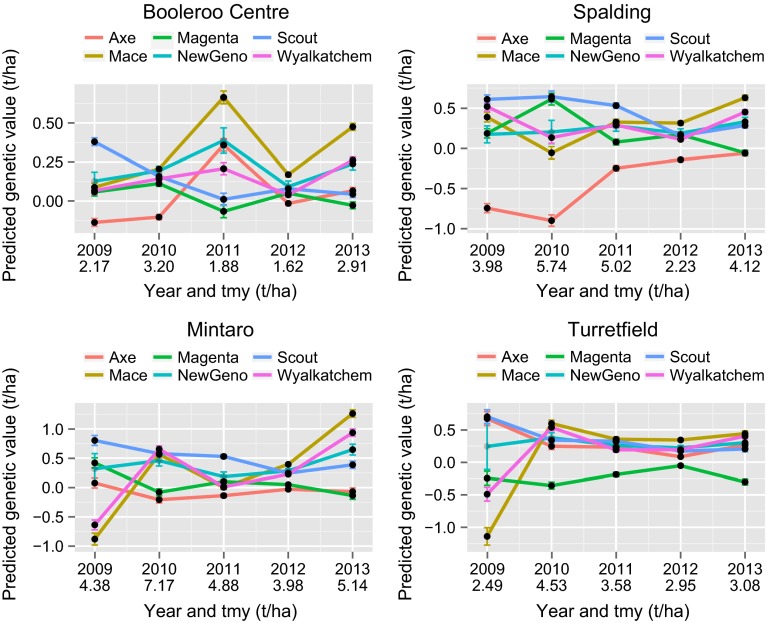
Predicted genetic values (t/ha), together with standard *error bars*, for six genotypes for environments in S3 region. The *panels* correspond to the four trial site locations used in this region and on each *panel*, predictions for an individual genotype are plotted against year of trial. *Points are coloured black/grey* if genotype was grown/not grown in the associated trial. The trial mean yield is shown on the $x$-axis underneath the year of the trial

Varietal stability as displayed in the latent regression plots is with reference to the entire set of environments under study. Growers may find it more helpful to limit the set of environments to those of specific interest. As previously discussed, grower information in Australia is currently disseminated on a regional basis. Given the familiarity of growers with this system we suggest graphical displays of the form in Fig. 11 which relates to the S3 region. The figure shows the predicted genetic values (and their standard errors) for the six varieties listed in Table 2 for the environments in which testing was conducted in the period under study. In this region, four trial sites were used, and there is a panel for each of these in Fig. 11. Within each panel, the predicted genetic values are shown for all six genotypes for all years in which the location was used. The points are coloured black or grey depending on whether the genotype was grown in the trial, and are joined between years by lines that are coloured differentially to identify the genotypes. The stability and high yielding performance of Scout is re-inforced on Fig. 11, with the variety ranking highest or near highest for all environments except Booleroo Centre in 2011. The variety Mace yielded highest in many environments, in particular at all locations in 2013 and at Booleroo Centre in 2011, but it yielded poorly at Mintaro in 2009 and Spalding in 2010 (both of these trials were affected by frost) and Turretfield in 2009 (this trial was affected by the disease stripe rust).

**Fig. 12:**
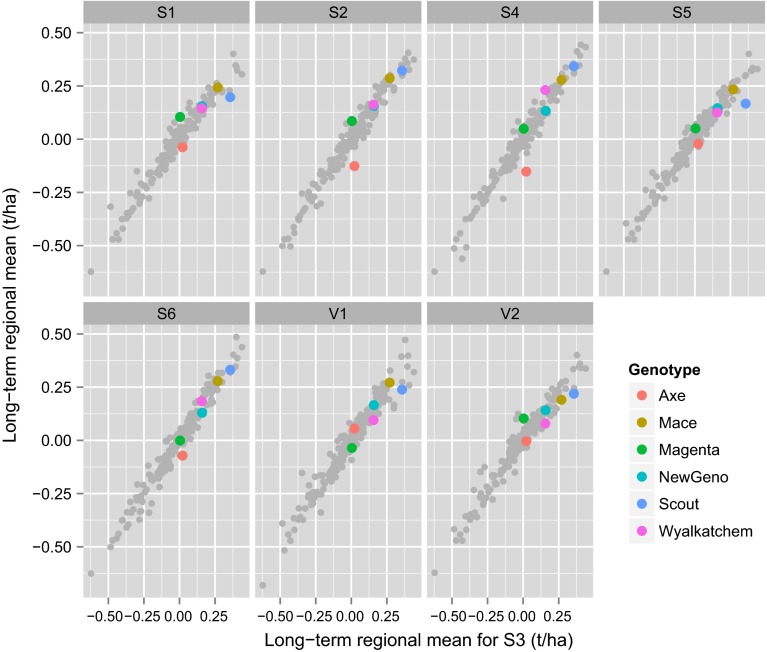
Long-term regional predictions for all varieties for individual regions, graphed against those for the S3 region. Six key varieties are highlighted

**Fig. 13:**
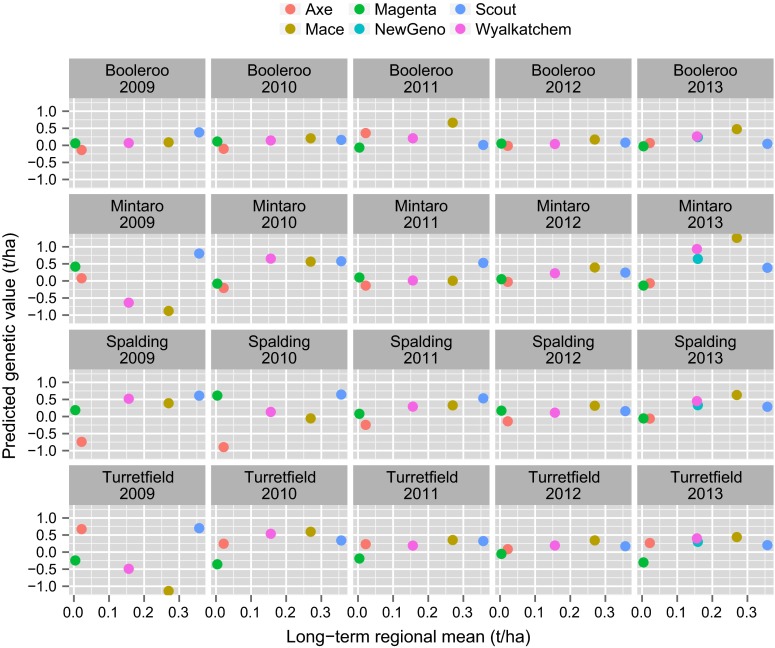
Predicted genetic values for 6 genotypes and 20 environments, graphed against the corresponding long-term regional predictions

It is instructive to compare variety predictions of the form presented in Fig. 11 with the long-term regional predictions historically provided. Firstly, recall that in the variance component analysis, the variety main effects and variety by region interactions accounted for 38 % and 4 % of the genetic variance, respectively. The lack of variety by region interaction is reflected in Fig. 12 in which long-term regional predictions for all varieties are graphed for each region against those from the S3 region, which is geographically central in the Southern mega-region. This figure shows that the use of long-term regional predictions means that all growers would be provided with very similar variety information, so that, on the basis of yield alone, variety selections would change very little across the entire Southern mega-region. At a within region level, by definition the long-term regional predictions are the same for all growers so that the presence of specific adaptation, for example, as discussed with reference to Fig. 11 is masked. This is shown in Fig. 13 in which the predictions in Fig. 11 are plotted against the corresponding long-term regional (S3) predictions.

**Fig. 14:**
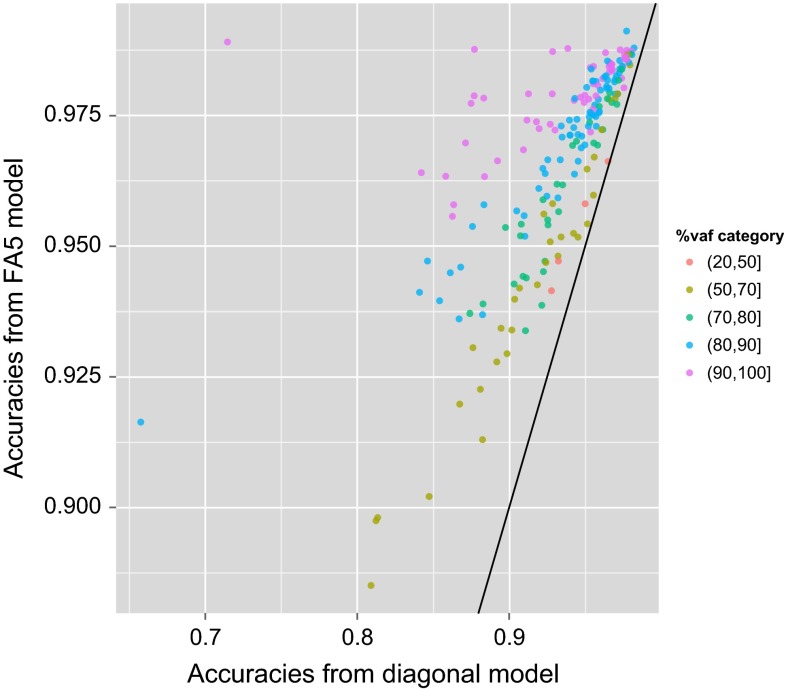
Accuracies of variety predictions from the FA5 and diagonal models. Each *point* relates to a different environment and is *coloured* according to the percentage of variance accounted for by the FA5 model

Finally, it is of interest to compare the (model-based) accuracy of variety predictions from the best-fitting FA model with those of the diagonal model. This allows an empirical examination of the impact of conducting a MET analysis in which genetic correlations are appropriately modelled. Accuracy may be computed, for each variety in an environment, as the correlation between the true and predicted effects, and computed from model output as in Mrode (2005). This was done for the total variety effects (that is, $u_{ij}$) for each environment from the FA5 and diagonal models. Environment values were then obtained by averaging accuracies across all varieties grown in each environment. Figure 14 shows that the accuracies of variety predictions for individual environments were always superior for predictions from the FA5 model compared with the diagonal model. Additionally, the gains were relatively greater for environments that were well explained by the FA5 model. That is, they had high $v_j$ values which also suggests they had strong genetic correlations with other environments under study.

## Discussion

CVT has been of fundamental importance in Australia for many years. Originally there were separate CVT programs in each state, but in 2005 the GRDC implemented the nationally co-ordinated system known as the NVT. The analysis of long-term MET data from these programs (both state-based CVT and more recently NVT) has followed the same approach since the mid 1990s, and is documented in Smith et al. (2001a). This involved the use of a variance component model with $V\times E$ interaction partitioned into sources associated with pre-defined geographic regions, locations within regions and years. The information reported to growers comprised long-term regional means, based on $V$ main effects and $V\times R$ [and possibly $V\times L(R)$] interaction effects.

The methodology of Smith et al. (2001a) is based on the classical approach of Patterson and Silvey (1980) which is still used in many countries, albeit with minor variations. After many years of experience with this approach in Australia, it became clear that there were inadequacies, both in terms of the goodness-of-fit of the model and the specificity of the variety predictions. There was a need for change, driven partly by statistical considerations, but more importantly by the concerns of growers and advisers. Given that these issues were central to the development of our new approach, we discuss them in detail in the following.

In terms of the model, a key issue is that the use of simple variance components imposes a very restrictive variance-covariance structure on the genetic effects. In particular, the genetic variance for every trial is assumed to be the same, and is given by the sum of the variance components. Given the large heterogeneity in error variance between trials in NVT MET datasets, it is likely that there is also heterogeneity of genetic variance. Similarly, the variance component model imposes restrictions on the pattern of genetic correlations between trials. In the extreme case of a linear mixed model in which $V\times E$ interaction is not partitioned, the resultant variance-covariance structure is known as a compound symmetric structure, and implies that the genetic correlation between all pairs of trials is the same. The partitioning of $V\times E$ interaction leads to some heterogeneity of genetic correlation, but it is very limited, with blocks of trials having the same correlation. The existence of variance and covariance heterogeneity in MET data and the inability of the variance component model to capture it, has been discussed by numerous authors, including Gogel et al. (1995), Smith et al. (2001b) and Piepho and van Eeuwijk (2002). Our experience in using FA models (Smith et al. 2001b) for MET datasets from plant breeding programs led us to consider the use of FA models in the NVT setting. There is substantial evidence in the literature that, for MET data, FA models are far superior to variance component models in terms of the goodness-of-fit (for example Smith et al. 2001b; Kelly et al. 2007; Mathews et al. 2007). This is true even when $V\times E$ interaction is partitioned as in Smith et al. (2001b) or Patterson and Silvey (1980), that is, into sources associated with regions, locations within regions and years. The results for the example presented in the current paper support this and have been replicated for the three other mega-regions for wheat and for all other crops in the NVT system (results not presented).

Initially we attempted to maintain the partitioning of $V\times E$ interaction as per the variance component model, and use FA variance-covariance structures for individual sources of $V\times E$. We encountered numerous difficulties, both computational and conceptual, with this approach. The computational difficulties associated with fitting multiple variance-covariance structures to MET data have been noted elsewhere. Piepho and van Eeuwijk (2002) attempted this for crop variety evaluation data using a so-called three-way model, with $V\times E$ interaction partitioned into $V\times L$, $V\times Y$ and $V\times L\times Y$. They commented that “Another problem is the great computational burden for fitting models with all three variance-covariance structures, especially when the structures are more complex than compound symmetric”. In the Australian setting, we consistently find that the two-way sources of $V\times E$ interaction, and in particular, the two-way static sources of $V\times R$ and $V\times L(R)$, are very small compared to the three-way interactions (Cullis et al. 2000). Thus there is insufficient information to allow the fitting of anything other than simple variance structures for the two-way interactions. An additional problem with multiple variance-covariance structures for nested terms in a mixed model, is the need to carefully consider identifiability and interpretation. The arguments for and against the use of a three-way structure in a MET analysis provides an interesting research problem, but for the reasons just described, we have chosen to use a two-way approach and fit a single FA variance-covariance structure for the $V\times E$ effects.

In terms of variety predictions, there has been mounting dis-satisfaction in Australia with the provision of long-term regional means. Growers and their advisors have had little confidence in these predictions as they know, from personal experience, that they fail to identify important local $V\times E$ interaction. Figures 12 and 13 clearly demonstrated the issue of long-term regional means being too “global”. Growers typically focus on one or two NVT trial locations they feel are similar to their own farm. They also take account of the season, so, for example, in a year in which they deem their chosen trial locations to have experienced atypical conditions, they would down-weight the results. There was statistical support for the growers’ concerns. Cullis et al. (2000) showed that $V\times E$ interaction for trials in the same region was often as large as that for trials in different regions and that this was primarily due to the fact that non-static $V\times E$ interaction, that is, as linked to seasonal influences is typically much greater than static interaction.

We note that some would argue that long-term regional predictions provide the most reliable information for growers. One of the key assumptions here is that the locations in the MET dataset comprise random samples of locations within regions, and that the years comprise random samples of seasons. In the Australian setting, locations are certainly not chosen at random, and as discussed above, growers are interested in specific locations (not necessarily in their own region). It is also arguable that a given sequence of, in our case, five years, constitutes a random sample of seasons. Another argument put forward for the use of long-term regional means is that only “repeatable” or static interaction can be exploited for variety recommendations. We do not agree, since, as discussed above, growers are interested in variety performance in individual years so they can separate typical and atypical seasonal conditions. We note, however, that if long-term regional predictions are desired, they can be obtained from the fitting of the FA model as simple averages of predictions across the relevant environments.

In the Australian context, the growers requirement for local variety predictions of relevance to their own farms, led them to use results from individual trial analyses to make varietal selection decisions. Such results are merely a snap-shot of what occurred on a specific area of land in a specific time-frame and are based on a very small amount of data (typically derived from three replicate plots for each variety). The results of individual trial analyses within NVT should be viewed as data for the MET analysis and not as information for selection decisions. In fact, if they are used for the latter, the risk of selection errors can be unacceptably high.

We therefore propose the reporting of predictions for the individual environments represented in the data-set, but as obtained from the fitting of the FA model. It is important to clarify that although environments are defined with reference to the trials in the data-set, that is, using the trial location name and year, they do not represent the trial itself (which is a past event) but rather represent all the environmental influences experienced during the conduct of the trial (which may be experienced again in the future). It is also important to point out that the predictions may be very different from the individual trial analysis results since they are based on substantially more data. Furthermore, they are based on an appropriate model for variety by environment effects, namely the FA model, that allows efficient estimation of the genetic correlations between pairs of environments. As a consequence, results from multi-variate theory imply that the accuracy of predictions for one environment are improved via genetic correlations with other environments [see for example Thompson and Meyer (1986)]. This was shown empirically for the example in Fig. 14.

Ideally there would be a range of environmental covariate information measured during each trial that could be used to characterise the environment. At present, such information is limited. We provide the trial mean yield as it may be useful in providing a quantitative grading of environments. We envisage that growers will proceed as they have previously done when using individual trial results. That is, they will choose trials from locations of interest to them, and consider predictions for a range of seasons. Formally the process may involve forming variety averages across these environments by assigning economic weights to the environments in much the same way as a selection index across traits would be formed for breeding purposes (Bernardo 2010). Environments that more closely reflect the grower’s target environment (Cooper et al. 1996), for example, in terms of geographic and/or seasonal characteristics, would receive greater weight. We do not presume to be prescriptive about weighting schemes since this will be grower specific and is beyond the scope of this paper. The key point is that, in contrast to the un-informed automatic averaging implicit in regional means from a variance component model, our proposal is to obtain meaningful averages based on the full $V\times E$ interaction in the data and knowledge of growers’ target environments. In terms of the reporting of variety predictions, graphical displays of the form given in Fig. 11 have been shown to a number of plant breeders and agronomists, all of whom agreed that this is an effective method of communication.

A limiting factor at present is that there is no predictive capacity for environments other than those in the data-set under study. The FA model allows the modelling of variety responses to environments that occurred, but cannot predict what would happen at a new location. This is still very useful information for growers, since they can weight this information according to the likelihood of those environmental conditions occurring at their location. Expanding the scope of prediction outside the data-set is the subject of current research. This is a very difficult task that requires the collation of a comprehensive set of environmental covariate information and the use of complex statistical models to capture potential non-linearity and interactions between covariates (also see Jarquin et al. 2014). To our knowlegde, no-one has successfully achieved prediction of variety differences using environmental covariates in the context of large systems like NVT.

Other areas of related, and current, research are the development of a statistical test to determine the appropriate order of factor analytic model; investigation of the minimum levels of connectivity required for reliable estimation of factor analytic variance parameters and modification of the mega-region approach to forming data-sets so that more data on newer varieties may be included.

### **Author contributions**

BC and AS developed the statistical approach. AS prepared the manuscript. AG prepared the data and conducted the factor analytic modelling. HK provided the motivation and encouragement to develop tools to explore variety by environment interaction in NVT data. All authors have read and approved the final manuscript.
